# Sepiolite-Hydrogels: Synthesis by Ultrasound Irradiation and Their Use for the Preparation of Functional Clay-Based Nanoarchitectured Materials

**DOI:** 10.3389/fchem.2021.733105

**Published:** 2021-08-13

**Authors:** Eduardo Ruiz-Hitzky, Cristina Ruiz-García, Francisco M. Fernandes, Giulia Lo Dico, Lorenzo Lisuzzo, Vanessa Prevot, Margarita Darder, Pilar Aranda

**Affiliations:** ^1^ Instituto de Ciencia de Materiales de Madrid (ICMM), CSIC, Madrid, Spain; ^2^ Centro de Investigaciones Energéticas, Medioambientales y Tecnológicas (CIEMAT), Madrid, Spain; ^3^ Laboratoire de Chimie de la Matière Condensée de Paris, Faculté de Sciences, Sorbonne Université, Paris, France; ^4^ IMDEA Materials Institute, Getafe, Spain; ^5^ Dipartimento di Fisica e Chimica – Emilio Segrè, Università degli Studi di Palermo, Palermo, Italy; ^6^ Université Clermont Auvergne, CNRS, ICCF, Clermont-Ferrand, France

**Keywords:** sepiolite, ultrasonication, gelation, hydrogels, xerogels, nanoarchitectures, clays, layered double hydroxides

## Abstract

Sepiolite and palygorskite fibrous clay minerals are 1D silicates featuring unique textural and structural characteristics useful in diverse applications, and in particular as rheological additives. Here we report on the ability of grinded sepiolite to generate highly viscous and stable hydrogels by sonomechanical irradiation (ultrasounds). Adequate drying of such hydrogels leads to low-density xerogels that show extensive fiber disaggregation compared to the starting sepiolite—whose fibers are agglomerated as bundles. Upon re-dispersion in water under high-speed shear, these xerogels show comparable rheological properties to commercially available defibrillated sepiolite products, resulting in high viscosity hydrogels that minimize syneresis. These colloidal systems are thus very interesting as they can be used to stabilize many diverse compounds as well as nano-/micro-particles, leading to the production of a large variety of composites and nano/micro-architectured solids. In this context, we report here various examples showing how colloidal routes based on sepiolite hydrogels can be used to obtain new heterostructured functional materials, based on their assembly to solids of diverse topology and composition such as 2D and 1D kaolinite and halloysite aluminosilicates, as well as to the 2D synthetic Mg,Al-layered double hydroxides (LDH).

## Introduction

Sepiolite as well as palygorskite are one-dimensional (1D) microfibrous silicates belonging to the clay minerals family. They exhibit unique structural and textural characteristics that determine their use in diverse applications from industrial adsorbents to rheological additives (Álvarez et al., 2011). Recently, sepiolite has sparked a renewed interest as a multifunctional material due to its use in the preparation of nanostructured organic-inorganic and inorganic-inorganic functional composites. The application of these materials is promising in fields as diverse as heterogeneous catalysis and bioreactors, bioplastics reinforcement and food packaging, supported membranes, functional nanopaper, carbon-clay composites for sensor devices, bionanocomposites for controlled drug delivery, non-viral DNA transfection and adjuvants of vaccines (Ruiz-Hitzky et al., 2011a; Ruiz-Hitzky et al., 2013; Ruiz-Garcia et al., 2014b; Ruiz-Garcia et al., 2014c; Alcântara et al., 2016; Castro-Smirnov et al., 2016; Ruiz-Hitzky et al., 2016; Castro-Smirnov et al., 2017; González del Campo et al., 2018; Lo Dico et al., 2019; Ruiz-Hitzky et al., 2019).

**FIGURE 1:**
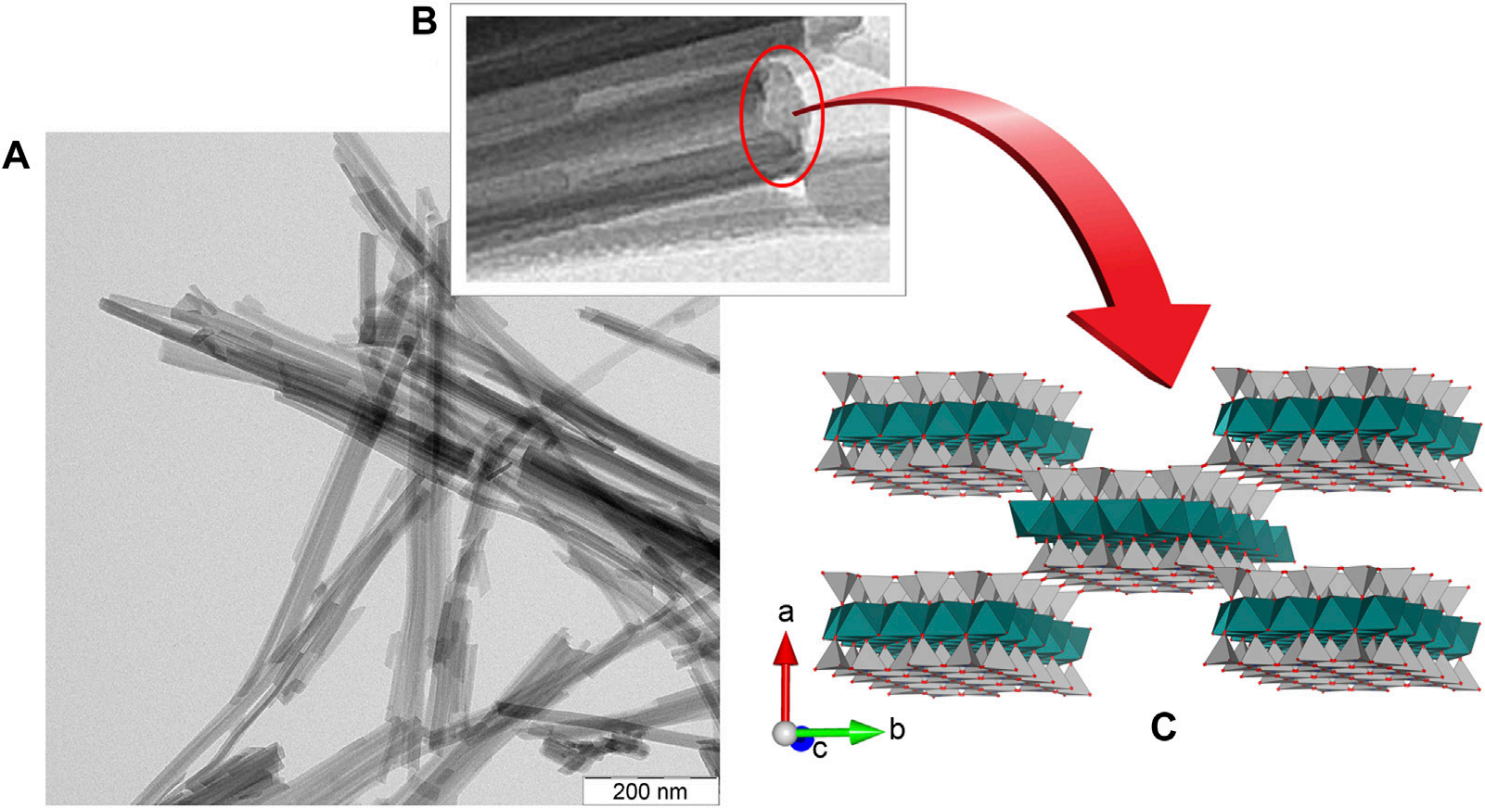
Fibrous morphology (TEM image) of **(A)** a sepiolite sample from Tagus Basin, Spain, **(B)** detail of a slightly tilted fiber, and **(C)** model of the crystal structure after Brauner and Preisinger (1956) represented with VESTA v3.1.8 software (Momma and Izumi, 2011).

Sepiolite is a hydrated magnesium silicate with Si_12_O_30_Mg_8_(OH,F)_4(_OH_2_)_4_·8H_2_O unit cell formula, its structural arrangement (Figure 1) consists in alternate Mg-silicate blocks (like in talc, i.e., tetrahedral-octahedral-tetrahedral sheets) and intracrystalline cavities named *tunnels* that grow in the crystallographic c direction, i.e., along the fiber direction. According to Brindley, the lath-like crystals of sepiolite develop on the (100) plane (Brindley, 1959). Tunnels that reach the external surface of sepiolite fibers can be considered as *channels*. These structures are involved in the interaction of many diverse compounds with the silicate, in particular through silanol groups (≡Si-OH) located at the edge of the channels (Ruiz-Hitzky, 2001). Other active sites at the sepiolite interface are the negatively charged surface attributed to isomorphical substitutions of Mg^2+^ ions by Al^3+^ and other trivalent cations in the octahedral layers. These two types of active sites are central to the adsorption mechanisms on fibrous clay minerals occurring at their external surfaces. However, structural tunnels also play an important role since they can act as molecular sieves, allowing for intracrystalline penetration of small size adsorbates as ions or neutral molecules, and exceptionally some linear polymers (Ruiz-Hitzky, 2001).

Suárez and García-Romero have proposed that sepiolite and palygorskite could be considered as the final members of a continuous polysomatic series (Suárez and García-Romero, 2013). Although the formation mechanism of these fibrous clay minerals remains unclear, most clues such as the very important deposits close to Madrid, at the Tagus basin in Spain (Leguey et al., 2010; Cuevas et al., 2011) as well as in the Serinhisar-Acıpayam basin of western Anatolia in Turkey (Akbulut and Kadir, 2003), point out to a sedimentary origin in lacustrine environments during the Miocene epoch. Leguey and co-workers described the genesis of sepiolite through biomineralization processes, showing that the textural organization of sepiolite fibers is quite similar to that of cellulose fibers produced by microorganisms (Leguey et al., 2014). Oriented aggregation of nanoparticles and mesocrystals formation for sepiolite and palygorskite clay minerals described by García-Romero and Suárez is compatible with their particular morphology. In fact, sepiolite “fibers” consist of prismatic crystals (laths) elongated along the [001] direction that aggregate parallel to the c-axis forming rods that are grouped together like fiber bundles (García-Romero and Suárez, 2014). These morphological characteristics, together with the physicochemical properties inherent to these silicates, are of utmost importance because they determine the ability of fibrous clay minerals to produce water-stable gels and, therefore, their role as stabilizers of various nanocomponents, leading to the production of a variety of architectured nanomaterials as studied in the present work.

Usually, sepiolite particles observed by electronic microscopy appear as bundles resulting from the agglomeration of the silicate microfibers. When sepiolite fibers are well dispersed in water they can form a colloidal network entrapping water molecules, producing high viscosity gels that are stable over time. Researchers at TOLSA company in Spain have pioneered the procedure of disaggregation of sepiolite bundles in concentrated water dispersions by applying high mechanical shearing, for instance by means of turbo-mixer machines (Alvarez Berenguer et al., 1985). This procedure preserves the aspect ratio of sepiolite fibers forming stable colloidal dispersions that, after drying by diverse techniques, result in “rheological grade” sepiolite products that are commercialized as thickening or suspending agents (e.g., Pangel^®^). By applying high-speed mechanical shearing (e.g., 12,000 rpm) to 3–6% w/w water dispersions of this rheological grade sepiolite, high viscosity gels (typically 20,000–24,000 cps at room temperature) are produced without perceptible syneresis phenomena (i.e., without exudation or expulsion of the liquid phase from the gel) at least for 72 h after preparation (Alvarez Berenguer et al., 1985). The dispersibility and the formation of stable suspensions of sepiolite can be significantly improved by incorporation of chemical dispersants such as polyacrylates, polyphosphates or carboxymethylcellulose (Alves et al., 2020). The disaggregation of the sepiolite bundles can also be performed by dry micronization of the previously dried raw material using a jet mill that generates micron-sized fibrous particles, which are marketed by TOLSA Company as Pansil. Compared to Pangel, which is prepared by wet disaggregation of sepiolite, Pansil produces dispersions with lower rheological performance, ultimately leading to other applications such as active fillers, anticaking agents and carriers in powder formulations (TOLSA_Group, 2020).

The use of high-frequency processing or ultrasonic vibrations is a powerful way to disperse and homogenize different solid particles dispersed in water (Furtado et al., 2004; Sauter et al., 2008). The attention received by this technique is also closely related to the advent of nanotechnology and nanoparticles. Due to the small dimensions of treated particles, and thus to their high surface to mass ratio, they show increased interfacial phenomena. For instance, ultrasonication of sepiolite in water dispersion strongly enhances its adsorption capacity attributed to the significant increase of the specific surface area, from ca. 300 to ca. 500 m^2^/g (Küncek and Şener, 2010).

Ultrasonication of clays is useful to disrupt aggregates, resulting in a significant reduction in particle size causing its de-agglomeration and forming gels with interesting rheological properties. This approach was initially applied for gelling sepiolite and palygorskite fibrous clays in the presence of fertilizers, to generate stable clay-fertilizers as hydrogels of interest for agricultural uses (Elrod Jimmie and Lee Robert, 1990; Elrod and Moore, 1990). Castillo et al., using ultrasonic cleaning equipment, have confirmed the formation of sepiolite hydrogels by a prolonged sonication treatment producing a high degree of entanglement of nanofibers that retain water molecules (Castillo et al., 2011).

An important progress related to the application of sonomechanical procedures refers to the multiwalled carbon nanotubes (MWCNT) stabilization in water in presence of sepiolite assisted by ultrasonic irradiation, leading to colloidal dispersions with different contents in MWCNT (Fernandes and Ruiz-Hitzky, 2014). Surprisingly, these two materials so different in nature, present a strong cooperative behavior leading to an enhanced colloidal stability in water, which can be interpreted in terms of steric stabilization. Highly hydrophilic sepiolite nanofibers act as the interposed species preventing the CNT re-aggregation (re-bundle), keeping them in a stable and homogeneous aqueous suspension (Fernandes and Ruiz-Hitzky, 2014). A similar procedure can generate stable dispersions of graphite (or graphene) nanoplatelets (GNP) (Ruiz-Hitzky et al., 2016), which also forms thin conductive films (*hybrid buckypapers*)—showing a planar arrangement of the GNP particles in this case. The assembly of MWCNT to these GNP dispersions drives to self-supported films with enhanced electrical conductivity. In addition, MWCNT/GNP-based bionanocomposites prepared under ultrasonic irradiation in presence of sepiolite are very promising for different applications as reported recently (Fernandes and Ruiz-Hitzky, 2014; Ruiz-Hitzky et al., 2016; González del Campo et al., 2018).

Various metal oxide nanoparticles (NP) have been assembled to sepiolite following co-precipitation procedures, hydrothermal synthesis, sol-gel methodologies, etc., giving rise to functional NP-sepiolite nanoarchitectures (Aranda and Ruiz-Hitzky, 2018). To achieve such materials, the clay should be effectively dispersed in the aqueous medium, with the fibers as untangled as possible. In this way, TiO_2_-sepiolite photocatalysts have been produced via sol-gel from titanium tetraisopropoxide in the presence of an organosepiolite dispersed under high shear (Aranda et al., 2008). This type of nanoarchitectured materials prepared via sol-gel involving silicon alkoxides also benefits from ultrasonic treatment to favor the disaggregation of the fibers as reported earlier (Gómez-Avilés et al., 2013). Ultrasounds can also be applied to produce the direct assembly of nanoparticles previously prepared to the clay, for example to prepare ZnO-sepiolite photocatalysts (Akkari et al., 2016). A clear example of how the aggregation of clay fibers influences the characteristics of the final material is the case of the *in situ* growth of layered titanosilicates (type JDF-L1) on sepiolite (Perez-Carvajal et al., 2019). In this example, the use of ultrasound to disperse the clay produces a greater disaggregation of the sepiolite fibers, which act as nucleation axis for the growth of the titanosilicate platelets, driving the growth of numerous nanocrystals of the titanosilicate from the fiber silicate. This is relevant because the final nanoarchitecture presents enhanced textural properties, placing these materials as interesting candidates for H_2_ storage among other applications.

**FIGURE 2:**
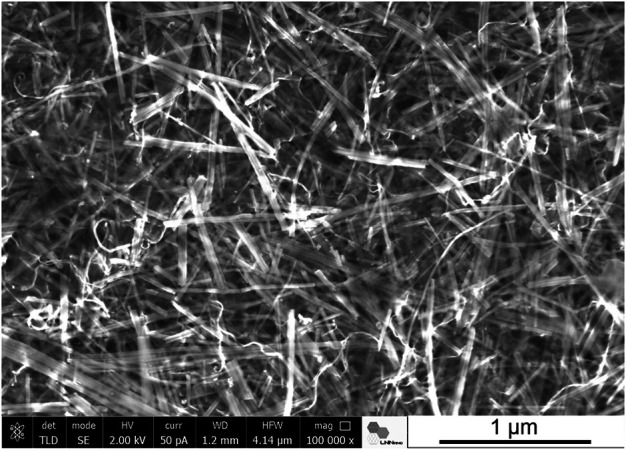
SEM image of a ternary system conformed as a self-supported film based on sepiolite fibers combined with cellulose nanofibers and MWCNT (González del Campo et al., 2018) (Image by J. Bettini, LNNano - CNPEM, Campinas, Brazil).

Ultrasound irradiation was also applied to produce nanocellulose/sepiolite nanocomposite materials using TEMPO-oxidized cellulose nanofibers (CNF) from eucalyptus wood (González del Campo et al., 2018). In this case, ultrasonication facilitated the assembly of both high aspect ratio components, resulting in homogenous hydrogels that yield very uniform nanopapers after drying. The incorporation of a third component to the CNF-sepiolite mixture, such as MWCNT, magnetite nanoparticles or ZnO nanoparticles gave rise to multifunctional materials featuring electrical conductivity, superparamagnetic properties or photocatalytic activity, respectively. Another example of the application of ultrasound irradiation in the production of nanocellulose/clay materials was the use of microcrystalline cellulose (MCC) as source of the cellulosic component (González del Campo et al., 2020). The ultrasounds contributed to produce defibrillation from the outer layers of MCC (Ching et al., 2018) giving rise to high aspect ratio cellulose nanofibers, and facilitated their combination with the sepiolite particles to yield homogeneous films, enabling the incorporation of additional components such as MWCNT (Figure 2). It is noteworthy that the assembly of the two hydrophilic fibrous components, CNF and SEP, leads to composite thin films that show hydrophobicity due to the surface roughness created by the hierarchical micro/nano structure of the assembly (González del Campo et al., 2018), which could be of interest for adsorption applications (Sanguanwong et al., 2021). Moreover, the assembly of halloysite nanotubes (HNT), which can be previously loaded with silicic acid and other molecular species, allows for the preparation of films for drug delivery applications (Lisuzzo et al., 2020b). Multicomponent conductive nanoarchitectured materials have been prepared by ultrasonication of sepiolite, HNT, GNP and chitosan. The inorganic components remain homogeneously integrated within the resulting composites exhibiting electrical conductivity together with specific adsorption properties, which is useful for advanced electrochemical devices such as biosensors and enzymatic biofuel cells applications (Lo Dico et al., 2019).

In this work, we unveil how ultrasonic treatments that promote the efficient dispersion of sepiolite fibers as well as other small-size components can be used to produce homogeneous and stable gels (hydrogels and xerogels) allowing for the preparation of diverse composites and nanoarchitectured functional materials. More specifically, we introduce here various examples showing how colloidal routes based on sepiolite gels can be used in their assembly to aluminosilicates such as kaolinite (2D) and halloysite (1D) clay minerals, and to a synthetic Mg-Al layered double hydroxide (LDH), to obtain the corresponding heterostructured clay functional materials.

## Materials and Methods

### Starting Materials and Reagents

In this study, two sepiolite (SEP) samples from Vicálvaro-Vallecas deposits, Madrid (Spain) supplied by TOLSA, S.A. (Madrid, Spain) were used: granular sepiolite with particle size of 0.250–0.600 mm, and S_BET_ surface area of 229 m^2^ g^−1^; and sepiolite of rheological degree with >95% purity commercialized as Pangel S9. It has a specific surface area (BET, N_2_) of 344 m^2^ g^−1^ and total pore volume of 0.4476 cm^3^. Some tests of preparation of SEP gels have been carried out using clay samples from Tagus basin in Spain (Sepiolite 30-60 and Sepiolite-1 furnished by TOLSA SA and SEPIOLSA, respectively) and from Balikesir in Turkey (Sepiolite-2 furnished by ZAFER MINING CO.). Some characteristics of these samples are shown in Supplementary Table S1.

Halloysite from New Zealand (NZCC product) and kaolinite (Kaolinite English China Clay product) were supplied by Imerys. Mg(NO_3_)_2_·6H_2_O (ACS reagent, 98%), Al(NO_3_)_3_·9H_2_O (ACS reagent, ≥98%) salts and NaOH (pellets EMPLURA^®^) were obtained from Sigma-Aldrich. The polysaccharide sodium alginate (ALG) (alginic acid sodium salt, from brown algae, with medium viscosity), was provided by Sigma Aldrich. Ultrapure water (resistivity of 18.2 MΩ cm) was obtained with a Maxima Ultra Pure Water system from Elga.

### Ultrasound-Assisted Production of Hydrogels and Xerogels

Sepiolite hydrogels were prepared from granular sepiolite without any previous treatment and dispersed in ultrapure water (100 ml) by tip-sonication (Sonics Vibracell VCX750 equipment with a Ti-Al-V tip of 13 mm diameter and operating at 20 Hz) in pulses of 10 s on/off until a total energy of 0.05–0.2 J/g or using a Hielscher equipment (UIP 2000hd) with a sonotrode (booster B2-1.8) operating in a flow cell with a recirculation system working until a total energy of 0.5–3.8 J/g.

The corresponding sepiolite xerogels were obtained by removing most of the water from sepiolite hydrogels (5 wt%, viscosity values between 10,000 and 15,000 cps) by: a) concentration by centrifugation at 9,500 rpm for 10 min and subsequent drying in air, b) drying by heating at 60°C under atmospheric pressure, c) vacuum filtration and drying at 25°C, d) by freeze drying (Telstar Cryodos -80) under dynamic vacuum at −80°C and 0.03 mbar after 24 h freezing, and e) by spray-drying (Büchi Mini Spray dryer B-290) operating in the open mode and using compressed air as the drying gas and a standard cyclone. The xerogels were then re-dispersed in water (100 ml) at 6 wt%, applying shear force (ULTRATURRAX^®^ T25 digital) at 12,000 rpm for 5 min to prepare the corresponding sepiolite hydrogels. These new hydrogels obtained by drying in different conditions were named as XG-CF, XG-60C, XG-VF, XG-FD and XG-SD, respectively, as indicated below.

### Preparation of Kaolinite-Sepiolite and Halloysite-Sepiolite Colloidal Dispersions and Self-Supported Thin Films

The kaolinite-sepiolite (KAOL-SEP) and halloysite-/sepiolite (HNT-SEP) suspensions were prepared at two different concentration values: 0.1 and 3 wt% of total solid in water. The required amount of Pangel S9 sepiolite (SEP) from TOLSA was dispersed in water and mixed with the correct amount of pristine HNT or KAOL. The resulting KAOL-SEP or HNT-SEP mixtures were tip-sonicated (Vibra Cell VC 750, 13 mm titanium sonication probe) until a total energy of 1 and 5 kJ was reached, respectively, using 10 s pulses separated by 10 s rest time (10 s on/10 s off). The same procedure was followed for the thin films preparation. Once prepared the suspensions at 0.1 wt%, the next step was the vacuum filtration, using 0.025 μm pore size filters (Millipore Membrane Filter, mixed cellulose esters, hydrophilic), which allowed the preparation of films with a thickness of about 0.050 mm. The samples were labeled as KAOL–SEP X-Y and HNT-SEP X-Y, where X and Y represent the mass percentage of KAOL or HNT and SEP respectively, and namely 0:1, 1:5, 1:2, 1:1, 2:1, 1:0 (w/w).

### Preparation of Layered Double Hydroxides-Sepiolite Nanoarchitectures and Derived Alginate-Based Nanocomposites

The Mg_2_Al(OH)_6_(NO_3_)·2H_2_O LDH (MgAl-NO_3_) was synthesized by flash co-precipitation (Xu et al., 2006a; Xu et al., 2006b; Pavlovic et al., 2017) followed by hydrothermal treatment. This procedure consisted in the rapid addition of 0.185 M NaOH solution to a solution containing magnesium and aluminum nitrates (Mg/Al = 2; 0.3 M) at 0°C. After adjusting the pH to 9.5, the resulting dispersion was transferred to an autoclave and heated to 150°C for 4 h. The obtained particles were collected by centrifugation, and after removing the supernatant the remaining gel was washed twice with deionized water, with re-dispersion achieved by ultrasonication between each washing step. Finally, the nanoparticles were re-dispersed in deionized water and stored as a colloidal suspension (10 wt%) at room temperature.

Water based SEP-LDH dispersions were prepared by adding 10 ml of ultrapure water to the appropriate amounts of clay and LDH, eventually followed by pulsed ultrasonic irradiation (VC750 Sonics Vibra-Cell, operating at 20 kHz) using a 13 mm standard probe. Typically, irradiation was carried out until an energy limit of 1 or 5 kJ, using 10 s pulses separated by 10 s rest time. Then the suspensions were filtered under vacuum Millipore system with a hydrophilic membrane (MF-Millipore, VSWP04700, pore Ø = 25 nm) and dried overnight at 40°C before being peeled-off for characterization. Note that pure LDH film cannot be peeled-off and form films.

SEP-LDH/ALG composites with several proportions of components were prepared by solvent casting from aqueous dispersions. Initially, the dispersions containing sepiolite and LDH in 5 ml of ultrapure water were submitted to ultrasonication treatment, using the same conditions indicated above for sepiolite and LDH dispersions. Then, 5 ml of alginate solution was added to each dispersion under magnetic stirring. In order to obtain the films, the sonicated dispersions were casted on a plastic Petri dish with diameter of 90 mm from Deltalabs S.L. and allowed to dry at 40°C. Detailed initial compositions for SEP-LDH and SEP-LDH/ALG films are reported in Table 1.

**TABLE 1:** Initial components in the mixtures for the preparation of SEP-LDH^a^ and SEP-LDH/ALG^b^ systems.

Sample	Components		
	Sepiolite (mg)	LDH (mg)	Alginate (mg)
SEP-LDH 5:1	100	20	0
SEP-LDH 1:1	20	20	0
SEP-LDH 1:2	10	20	0
SEP-LDH 1:0	20	0	0
SEP-LDH 0:1	0	20	0
ALG	0	0	40
SEP/ALG 1:1	20	0	20
LDH/ALG 1:1	0	20	20
SEP-LDH/ALG 1:1:2	20	20	40

a Mixtures treated at 0, 1 and 5 kJ.

b Mixtures treated at 1 kJ.

### Characterization Techniques

XRD patterns were obtained on a BRUKER D8-ADVANCE diffractometer, using Cu Kα radiation. The voltage and current sources were set at 40 kV and 30 mA, respectively. Diffraction patterns were recorded with a goniometer speed of 0.5 s per step between 4° and 70° (2*θ*). In the case of LDH materials, the XRD patterns were collected with a PANalyticalX'Pert Pro diffractometer equipped with an X’Celerator Scientific detector and a Cu anticathode (Kα1/Kα2). The diffracted beam was detected over a range of 5°–70° (2*θ*) with a step size of 0.0167° and a counting time of 350 s/step. FTIR spectra were obtained with a Bruker iFS 66VS spectrophotometer with 2 cm^−1^ resolution. The dichroic effect in KAOL-SEP and HNT-SEP films was examined by tilting the film sample respect to the IR beam from 0° to 45°. In the case of halloysite/sepiolite materials, a test with DMSO was performed exposing the HNT-SEP 1:2 film in DMSO vapor for 24 h. Then, the sample was taken and immediately the FTIR spectra and XRD patterns were recorded. The FTIR spectra of the LDH materials were acquired in the attenuated total reflectance (ATR) mode using a Spectrum 100 FT-IR spectrometer (PerkinElmer). The solid material was placed on the surface of an internal reflection unit (previously washed with ethanol, ultrapure water and dried) made of a diamond crystal. The spectra were recorded in the range between 4,000 and 400 cm^−1^ at a resolution of 4 cm^−1^. A ZetaNano ZS (Malvern) device was used to determine the electrophoretic mobility and a compact goniometer system (ALV/CGS-3) was used for the dynamic light scattering (DLS) measurements. Water content of the xerogels was determined from thermogravimetric analyses (TGA) performed with a Setaram TGA92 thermogravimetric analyzer in the temperature range of 25–150°C, with a heating rate of 5°C min^−1^, under air atmosphere. For the HNT-SEP and KAOL-SEP samples SEM analysis was performed, after Cr metallization, by using a Philips XL 30 S-FEG Field Emission Scanning Electron Microscope. Film cross-sections were obtained by cutting the films after immersion in liquid N_2_. In the case of SEP xerogels and LDH-SEP samples, FE-SEM images were obtained with a NOVA NanoSEM230 microscope from FEI, with direct observation of the films adhered on a carbon tap and without any conductive coating on their surface. LDH nanoparticles were also observed by transmission electron microscopy (TEM) using a Hitachi 7650 microscope at an acceleration voltage of 80 kV. To perform the characterization, a drop of the suspension was deposited on a 400 mesh holey carbon-coated copper grid and dried at room temperature. TEM images of the sepiolite xerogel were obtained in a STEM LEO 910 apparatus operating at 120 kV. The volume of micropore (V_micro_) and external surface (S_EXT_) were determined by t-plot method, the specific surface area (S_BET_) was obtained by the Brunauer–Emmett–Teller method and mesopore (V_meso_) was calculated substracting the V_micro_ to the total volume adsorbed, from data of nitrogen adsorption/desorption isotherm measured at 77 K in ASAP 2420 (Micromeritics) system after degassing at 120°C. Viscosimetry measurements were done in a RVDVII + PRO Brookfield using the RV5 and Helipath-E spindle at 100 and 5 rpm of speed and at the temperature of 21 ± 0.2°C.

## Results and Discussion

### The Production of Sepiolite Gels by Ultrasonication

As indicated above, sepiolite water suspensions show great interest to assemble the clay mineral with both organic and inorganic materials, giving rise to a wide variety of functional heterostructures. We have also indicated that very stable aqueous suspensions of sepiolite could be achieved by decreasing the degree of natural agglomeration of its fibers by grinding that clay in dry or humid conditions, which entails a reduction in particle size and help to form the stable hydrogels. Commercialized products for uses as thickener, gellant or suspending agent are mainly based on the EP0170299A2 patent (Alvarez Berenguer et al., 1985). As considered here, an alternative procedure able to produce hydrogels and their corresponding xerogels by further drying processes, could be achieved by direct gelation of the sepiolite using ultrasound irradiation (Ruiz-Garcia et al., 2014a).

In this work we report on the easy disaggregation of sepiolite fibers applying sonomechanical treatments of sepiolite powder suspended in water leading to high viscosity and stable hydrogels without the use of any dispersant agent. It is known that ultrasonic irradiation has two main action modes: i) the solvent (water in the present case) generates cavitation bubbles due to the high pressure difference of shock waves and micro-jets when cavitation bubbles collapse, and ii) the absorption of ultrasonic energy by the liquid medium that also induces a high mechanical shear on this environment (Mamvura et al., 2018). In this context, ultrasonication of sepiolite/water mixtures can strongly determine the de-agglomeration of the natural sepiolite bundles that makes difficult the fibers disaggregation driving to stable colloidal systems. The rheological characteristics, such as the stability and viscosity of these dispersions, depend on several factors, such as the chemical and mineralogical composition of the starting clay, the concentration of dispersed solids, the starting particle size, and the applied energy.

Unlike sepiolite, which results in highly viscous stable hydrogels, attempts to generate clay hydrogels by applying similar ultrasound treatments using palygorskite lead to inhomogeneous dispersions with very low viscosity. Recently, Ferraz and co-workers (Ferraz et al., 2021) have reported on the production of relatively stable palygorskite dispersions by ultrasound irradiation, although these authors point out the requirement to incorporate dispersants such as polyphosphates to obtain better dispersions of this silicate. The different behavior observed between sepiolite and palygorskite to give viscous and stable gels is not clear. Perhaps the impurities that accompany palygorskite could negatively affect its ability to produce hydrogels under ultrasound irradiation. Therefore, the present work focused exclusively on sepiolite gels.

### Sepiolite Hydrogels by Ultrasonication

**FIGURE 3:**
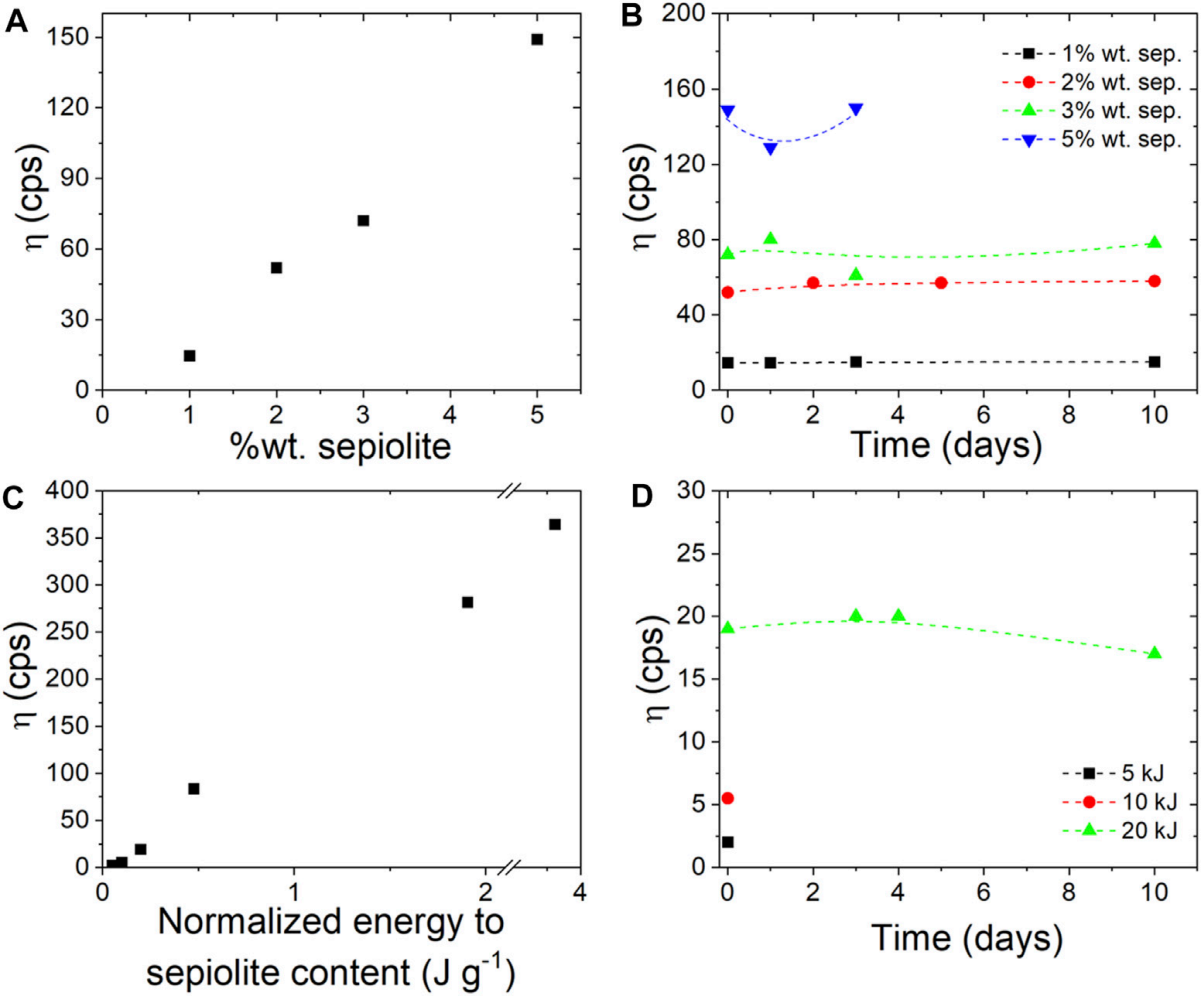
Brookfield viscosity of hydrogels measured using RV5 at 100 rpm: **(A)** of hydrogels obtained with different sepiolite content and applying the same energy value (20 kJ), **(B)** at different times of the hydrogels prepared using 1, 2, 3 and 5 wt% of sepiolite content and applying 20 kJ of energy, **(C)** after the sonomechanical irradiation of sepiolite clay at different energies, and **(D)** of hydrogels obtained from dispersion of 1 wt% sepiolite and applying 5, 10 and 20 kJ of sonomechanical energy.

In this study, a commercial granular sepiolite was treated by ultrasonic irradiation to generate stable colloidal dispersions as indicated in the Experimental Section. The concentration of the clay should be higher than the percolation threshold, i.e., the minimum concentration to obtain the dispersion as a single and continuous phase. Therefore, the rheological behavior of sepiolite suspensions in water directly depends on the sepiolite concentration (Figure 3A). As expected, higher viscosity values were obtained by increasing the sepiolite content, achieving values from 14 cps for 1 wt% clay contents to 149 cps when the clay content was increased to 5 wt% (operating at 0.2 J/g in static sonication mode). The stability of the hydrogels prepared is considered satisfactory, especially for sepiolite dispersions that were prepared using 2–3% solids without any sign of syneresis up to at least 10 days (Figure 3B).

The viscosity values stabilize in all the experiments carried out adopting the same conditions except the sepiolite concentration, although it could be considered that the resulting values for dispersions as low as 1 wt% sepiolite did not show practical interest for viable applications. In addition, the rheological behavior of sepiolite suspensions depends on the energy applied during ultrasonic irradiation, since the wet grinding that causes de-agglomeration of the fiber bundles can be more intense at higher sonication energy values. Hence, the application of higher energy values in the ultrasonic irradiation process will lead to more viscous clay dispersions (Figure 3C). However, it is important to note that a minimum energy is necessary (*ca.* 0.2 J/g) to sufficiently de-agglomerate the fiber bundles and consequently to achieve stable colloidal dispersions, otherwise the viscosity values achieved would be very low, appearing syneresis effects. Stable viscosity values, showing no sign of syneresis even up to 10 days after dispersion, are reached by applying energies of up to 0.2 J/g (Figure 3D).

To check the possible influence of the starting sepiolite characteristics on the efficiency towards the fibers disaggregation, several samples of different origin have been included in this study (see Experimental Section). It is observed that sepiolite samples from the Tagus basin in Spain reach higher Brookfield viscosity values (60–109 cps) than sepiolite from Turkey (40 cps) under the same experimental conditions. However, regarding the nature of the initial samples, there is no clear evidence of what factors can control the disaggregation of the silicate bundles and the subsequent generation of stable and viscous gels. According to Alvarez Berenguer et al. (1985), only high purity sepiolite samples (>80% pure sepiolite content) with a low smectite content (<10%) are required for a convenient development of gels by applying the patented wet shear procedure leading to the commercial rheological grade sepiolite products (Alvarez Berenguer et al., 1985). The three samples selected here show high purity in sepiolite (Table 1) but they give gels of different viscosity following the procedure based on ultrasonication of granular sepiolite dispersions here studied. It is only observed a higher content in iron oxide in the sample from Turkey compared to sepiolites from the Tagus basin (Supplementary Table S1) but any relationship between this feature and the gelling of the clays can be identified. In order to know the optimal conditions required to produce stable viscous gels, in addition to the chemical and mineralogical composition, it seems necessary to enlarge the studies on the influence of crystallinity, textural properties, fiber dimensions and zeta potential, among other characteristics of the starting samples.

### Sepiolite Xerogels: Characterization and Properties

The sepiolite xerogels prepared in this work result from diverse drying processes of the above synthetized hydrogels by application of the following approaches:

1) concentration by centrifugation and subsequent drying in air (*XG-CF*)
2) water evaporation by heating at 60°C under atmospheric pressure (*XG-60C*)
3) vacuum filtration and subsequent drying in air (*XG-VF*)
4) freeze drying (*XG-FD*)
5) spray-drying (*XG-SD*)

The water content of resulting xerogels depends on the selected drying procedure, being 18.0 and 17.5% for freeze drying (*XG-FD* samples) and drying at 60°C (*XG-60C* samples) procedures, respectively. Slightly minor water contents correspond to xerogels prepared by air-drying after vacuum-filtration (*XG-VF* samples) or after centrifugation (*XG-CF* samples), in these cases showing water contents of 13.6 and 13.4%, respectively.

**FIGURE 4:**
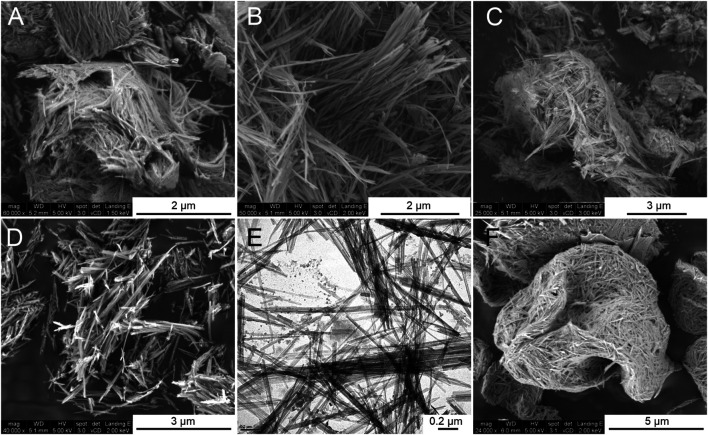
FE-SEM images of **(A)** starting 30–60 granular sepiolite, and sepiolite xerogels obtained by **(B)** freeze-drying, **(C)** centrifugation and air-dried, **(D)** drying by evaporation at 60°C, and **(F)** spray-drying. The image **(E)** corresponds to TEM micrograph of xerogel obtained by evaporation at 60°C.

The effect of ultrasonic irradiation is the key to obtain a high degree of wet disaggregation of the sepiolite fibers that give rise to hydrogels, but the textural characteristics of the derived xerogels also depend on the type of drying treatment applied. The FE-SEM images of the starting sepiolite and some of the prepared xerogels show morphological differences mainly related to the state of aggregation of the silicate fibers. Thus, Figure 4A shows the morphology corresponding to the 30–60 granular sepiolite used here as the starting material, showing the typical compacted bundles that make up the sepiolite fibers as they are present in the mineral deposits. The sepiolite xerogel obtained by evaporation of water in air after centrifugation of the sepiolite hydrogel produces a degree of defibrillation as shown in Figure 4C. The disaggregation of the clay bundles is well evidenced after freeze-drying (Figure 4B) or drying by evaporation at 60°C of hydrogels (Figure 4D). In both cases, the resulting xerogels do not exhibit the typical bundles of the starting sepiolite. Concerning the spray-drying technique applied to dry the sepiolite hydrogel shows a resulting reorganization of pseudo-spherical compacted skeins of the fibers as observed in the corresponding FE-SEM images (Figure 4F). On the other hand, TEM micrographs show individualized sepiolite fibers for the XG-60C samples as observed in Figure 4E. This image is quite similar to that corresponding to commercial Pangel S9 rheological product from TOLSA SA [see for instance TEM images in Aranda et al. (2008) or Gómez-Avilés et al. (2013)], which is also prepared following a wet disaggregation procedure (Alvarez Berenguer et al., 1985). Furthermore, it is important to note that no relevant fractures of the clay fibers are detected from the micrographs after the ultrasound irradiation treatments.

**FIGURE 5:**
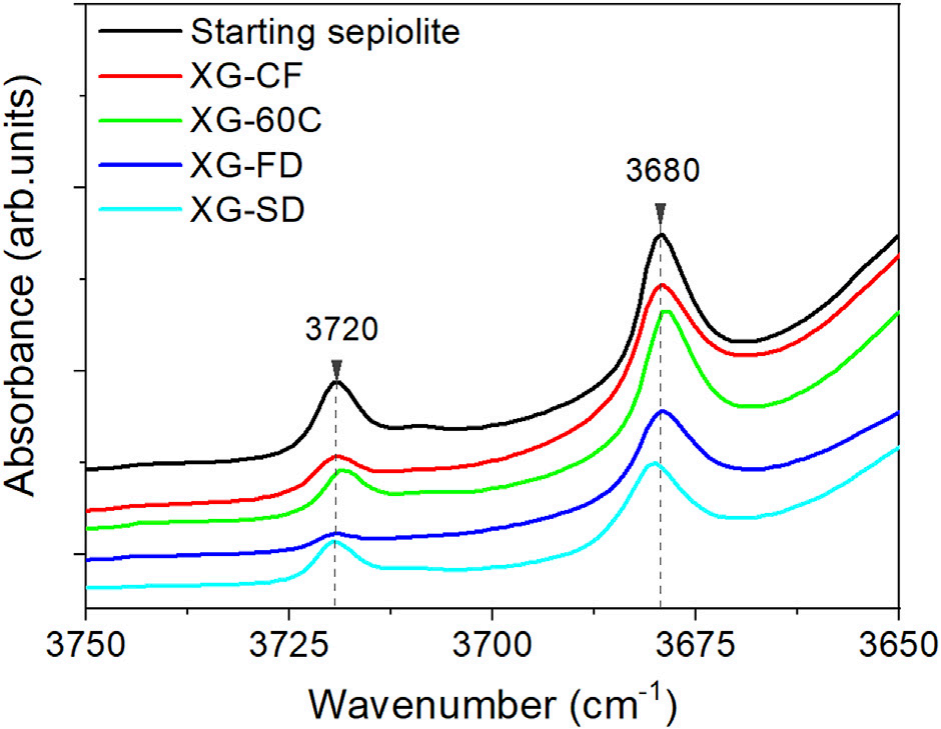
FTIR spectra (3,750–3,650 cm^−1^ region) of the sepiolite xerogels.

The nitrogen adsorption-desorption isotherms of the diverse prepared xerogels obtained at 77K (Supplementary Figure S1) exhibit a quite similar shape, which can be identified as type I at low relative pressure values in agreement with the characteristic microporosity of the sepiolite samples, and as type IIb at medium and high relative pressures with a narrow hysteresis loop indicating an interparticle capillary condensation. Values of BET specific surface area, external surface (S_EXT_), micropore volume (V_micro_) and mesopore volume (V_meso_) calculated from these isotherms are shown in Table 2. It can be observed that the values of BET specific surface are in the 118–275 m^2^/g range for the different samples depending on the adopted drying procedure. Therefore, the water elimination driving to the fibers re-agglomeration has a significant dependence on the adopted experimental conditions. It is also observed that the external surface values are in general less affected, being in the 102–135 m^2^/g range. Regarding the volume of pores attributed to the range of micropores, it also varies as a function of the applied drying process. The observed decrease is especially evident for samples also showing a decrease with respect to the starting sepiolite as this micropore volume largely contributes in the measured total surface area. As the micropores are mainly related to the structural tunnels of sepiolite, it could be admitted that a partial blockage of the micropores occurred during the disaggregation process of the fibers. This could be tentatively explained by admitting that such processes, taking place in an aqueous medium with the involvement of intense sonomechanical energy, could produce a leaching of the Mg^2+^ ions located at the edges of the sepiolite channels. We suggest here that a low amount of lixiviated magnesium hydroxide could thus accumulate at the surface of the silicate, preventing the access to the structural tunnels of the N_2_ molecules used as probe to obtain the adsorption isotherms. In fact, it has been already observed that a non-negligible fraction (<10% w/w) of Mg^2+^ cations and hydroxyl groups coordinated to them in the octahedral layers of sepiolite are spontaneously lixiviated when the silicate dispersions are maintained in water under continuous agitation at room temperature (Casal and Ruiz-Hitzky). To support this interpretation, it could be considered the observed relative decrease in the intensity of the O-H stretching vibrations of the Si-OH groups located at the external sepiolite surface, which appear in the IR spectrum at 3,720 cm^−1^, with respect to the O-H vibrations of Mg-OH located in the octahedral layer of sepiolite appearing at 3,680 cm^−1^ (Figure 5). Anyway, the structural alteration of the initial crystallinity of sepiolite is apparently maintained after the ultrasound treatment, according to the unchanged width of the diffraction peaks in the XRD patterns. On the other hand, the application of ultrasound is capable of producing nanometric individual fibers dispersions with a particle size distribution determined by TEM, which has been already detailed in a previous work (Castro-Smirnov et al., 2016).

**TABLE 2:** Values of BET specific surface area, external surface area (S_EXT_), micropore volume (V_micro_) and mesopore volume (V_meso_) deduced from the N_2_ adsorption-desorption isotherms of xerogels samples prepared by different drying procedures.

Sepiolite samples	S_BET_ (m^2^ g^−1^)	S_EXT_ (m^2^ g^−1^)	V_micro_ (cm^3^ g^−1^)	V_meso_ (cm^3^ g^−1^)
Starting sepiolite^a^	229	108	0.048	0.3126
XG-CF	275	136	0.055	0.3154
XG-60C	176	111	0.026	0.2659
XG-FD	118	102	0.005	0.3208
XG-SD	180	123	0.022	0.3033

a 30/60 granular sample.

The aggregation degree of sepiolite fibers is determinant with respect to the rheological properties of the samples after its re-dispersion in water under mechanical shear. The new hydrogels obtained give rise to stable colloids with very high Brookfield viscosity values at 6% w/w of xerogel measured at 5 rpm and using the Helipath T-E, being 20,000, 23,000 and 31,000 for XG-CF, XP-VF and XG-60C and XG-FD, respectively. These values can be comparable to the values of the rheological sepiolite commercialized with the trade name of Pangel S9, showing typical viscosity values between 21,000 and 34,000 cps (Alvarez Berenguer et al., 1985). Some of the resulting hydrogels prepared by re-dispersion of the here prepared xerogels exhibit an extraordinary stability without syneresis effects, even almost 10 years after its preparation (see Supplementary Figure S2).

### Sepiolite-Kaolinite and Sepiolite-Halloysite Nanoarchitectures

Both, kaolinite and halloysite are 1:1 layered dioctahedral aluminosilicates of Al_2_Si_2_O_5_(OH)_4_ chemical formula, each layer being structurally composed by one tetrahedral silica sheet linked to one octahedral aluminum-oxyhydroxide sheet (Ross and Kerr, 1931; Bauluz-Lazaro, 2015). Halloysite could include a monolayer of water covering the internal surface of each layer and therefore, considering the thickness of the H_2_O molecules (ca. 0.28 nm), its d_L_ basal space is higher than that of kaolinite, i.e., ca. 1.00 nm instead ca. 0.72 nm, respectively, when halloysite is hydrated. Kaolinite shows a plate-like morphology (2D), whereas certain samples of halloysite, as it is the one used in this work, show a characteristic nanotubular aspect (1D). Both clay minerals are usually present in nature as micron or submicron sized particles (Bauluz-Lazaro, 2015). External and luminal diameters of halloysite range from 20 to 150 nm and from 10 to 15 nm, respectively (Lisuzzo et al., 2021). Kaolinite and halloysite exhibit a limited capacity to intercalate small neutral polar molecules (e.g., urea, N-methylformamide, DMSO), which can be retained between the silicate layers, although halloysite can additionally insert diverse components inside the core lumen. This feature is particularly useful for subsequent controlled release of active agents (Lisuzzo et al., 2019b; Lo Dico et al., 2019; Lisuzzo et al., 2020a; Farinmade et al., 2020; Saleh et al., 2020; Stavitskaya et al., 2020). Moreover, this clay does not show toxic effects and it is biocompatible (Fakhrullin and Lvov, 2016; Rozhina et al., 2020; Rozhina et al., 2021).

One of the main limitations in the use of kaolinite and halloysite is the instability of their aqueous dispersions. The stability of kaolinite in water depends on the pH and the concentration of electrolytes (Chow, 1991). The low stability of these suspensions, particularly at relatively elevated concentrations, could be significantly increased by adding surfactant agents such as sodium dodecyl sulfate (SDS). Stabilization of halloysite water dispersions could be also improved by using surfactant agents (Lisuzzo et al., 2019a), as well as diverse polymers such as chitosan, pectin and hydroxypropyl cellulose (Bertolino et al., 2017). It has been proposed that a steric stabilization mechanism may be responsible in these cases. From a fundamental point of view, steric stabilization consists on the enhancement of the repulsive forces between two adjacent particles that hold an adsorbed species on its outer surface, thus augmenting the net stability of the colloid (Fernandes and Ruiz-Hitzky, 2014; Cavallaro et al., 2016). In this way, the aggregation of clay particles mainly produced by van der Waals attraction and through hydrogen bonding interactions could be effectively hindered by the presence of polymer chains between the involved solids (Lisuzzo et al., 2018).

As an alternative to the abovementioned approaches, in this section, sepiolite fibers have been incorporated to kaolinite or halloysite dispersions following a process assisted by ultrasonic irradiation with the aim to act as interposed species, allowing to reach colloidal dispersibility and stability in aqueous media in the long-term. The resulting sepiolite-kaolinite and sepiolite-halloysite dispersions lead to the corresponding clay based-nanoarchitectures after recovering and drying of these solid particles. It is worth to note that self-supporting films are created by using only inorganic components, belonging to the class of clays, without any addition of polymers.

**FIGURE 6:**
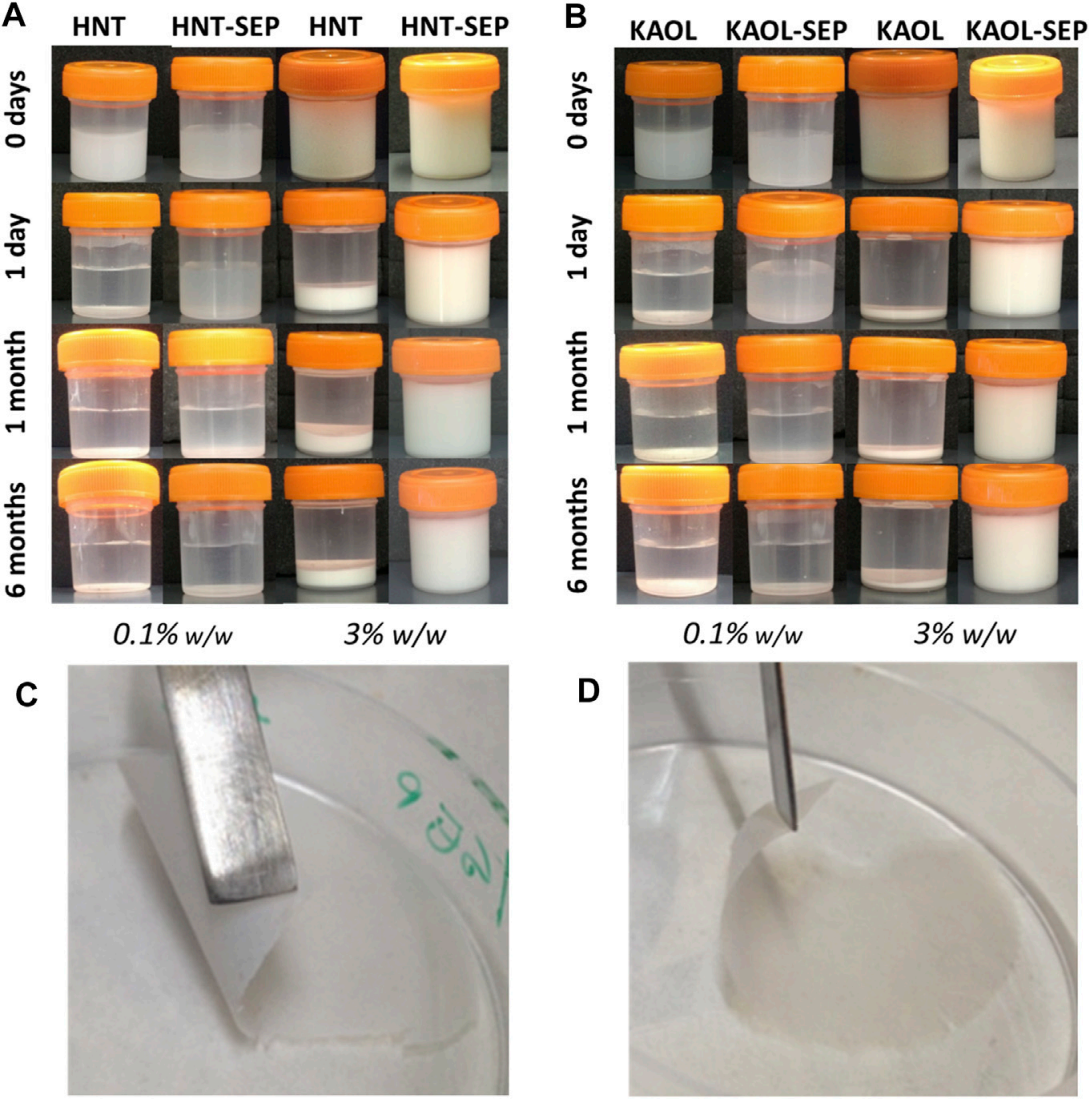
Macroscopic aspect of 0.1 and 3.0 wt% aqueous dispersions of **(A)** pristine halloysite and HNT-SEP (1:5); **(B)** pristine kaolinite and KAOL-SEP (1:5); and picture of self-supported **(C)** HNT-SEP 1:5 film and **(D)** KAOL-SEP 1:5 film.

Concerning the instability of the water dispersions of kaolinite and halloysite after ultrasonication, it is observed that both the 0.1 and 3 wt% dispersions of both pristine clay minerals rapidly separate into solid/liquid phases after less than 1 day (Figure 6). However, upon sonication after the addition of sepiolite to each of the aluminosilicate clay dispersion, the resulting gels are stable in both cases (sepiolite-kaolinite and sepiolite-halloysite). The formation of these stable and homogeneous gels could be explained assuming the sepiolite fibers create an intricate network that would be responsible for the generation of a steric obstacle, hindering the re-bundling and consequently the flocking of the solids from the water dispersion. The optimization of the stabilization conditions was first carried out considering the amount of ultrasonic energy required to disperse the involved clays. The optimal experimental conditions to maintain very stable gels without syneresis effects correspond to an irradiated energy between 1 and 5 kJ. Images in Figures 6A,B show that both 0.1 and 3 wt% HNT-SEP and KAOL-SEP dispersions remain stable and homogeneous at least after 24 h since their preparation.

It is noteworthy that, while the 0.1 wt% KAOL-SEP and HNT-SEP dispersions separate after 1 month, the 3 wt% dispersions remain stable and homogeneous even after a long period of observation, namely at least 6 months after preparation. These results can be explained considering the rheological percolation threshold of sepiolite particles in water, which corresponds to ca. 0.1 wt% in pure sepiolite (Fernandes and Ruiz-Hitzky, 2014). Thus, when the dispersions are prepared with concentrations in sepiolite below this limit, they tend to segregate into two phases (particle-free and particle-rich phases). On the other hand, when the amount of involved sepiolite is higher than that limit, the dispersion is stable without syneresis effects, confirming that sepiolite is able to create and maintain a fibrous network that prevents the re-aggregation of nanotubular (HNT) or lamellar (KAOL) particles, therefore keeping these systems well dispersed. These observations agree with what was already described for sepiolite-MWCNT systems (Fernandes and Ruiz-Hitzky, 2014).

**FIGURE 7:**
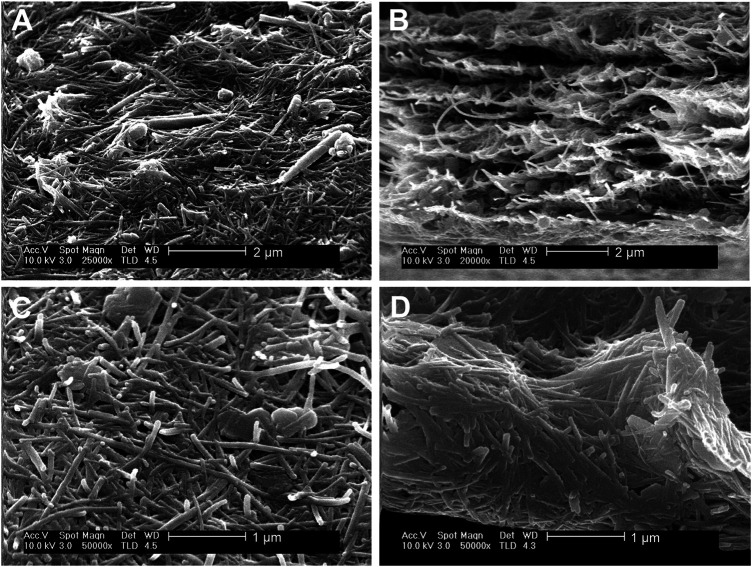
SEM micrographs of HNT-SEP 1:1 **(A)** surface and **(B)** cross section, and of KAOL-SEP 1:2 **(C)** surface and **(D)** cross section.

An important conclusion of these homogeneous co-dispersions of the clays is that they can be used to produce self-supported thin films of the clays after controlled elimination of water. The resulting films cohesion depends on the strong assembly of sepiolite nanofibers with platelets or nanotubes without the involvement of any polymer or surfactant agent. Indeed, to the best of our knowledge this is the first time that solely clay-based and fully inorganic self-supported and flexible films containing kaolinite or halloysite have been reported. The highly homogeneous macroscopic aspect is observed in Figures 6C,D. FE-SEM images of the surface of the films and their cross-section (Figure 7) show the presence of the layered kaolinite and nanotubular halloysite particles embedded within the network created by the sepiolite nanofibers.

XRD patterns of the films (c.f. HNT-SEP 1:2 and KAOL-SEP 1:2 films in Supplementary Figure S3) confirm that the structures of the involved clays remain unaltered. For what concerns HNT-SEP films, the unchanging in the typical reflection of (100) plane (d = 0.73 nm) of halloysite, i.e., without evidence of new reflection peak at lower 2*θ* value, suggests that the HNT remains in its dehydrated form and no intercalation occurred (Rooj et al., 2010; Bertolino et al., 2016). The absence of intercalation is important in view of further uses of halloysite as nanoreactor, nanocontainer or microenvironment to preserve molecules inside the lumen of the HNT.

The analysis of the XRD patterns also provides a first evaluation of textural features of the prepared self-supported films. Generally, the (100) diffractions are dominant when fibers, nanotubes and sheets are ideally oriented lying on the plane of the film (on b- and c-axes to make drawings), while the other diffraction peaks display weaker intensity (Umemura et al., 2009; Żbik et al., 2010; Ruiz-Hitzky et al., 2011a; Pasbakhsh and Churchman, 2015). On this basis, the intensity ratio (100)/(020), as well as (200)/(020), were chosen as parameters to define the orientation of kaolinite sheets and halloysite nanotubes, meanwhile the (200)/(110) and the (400)/(110) ratio of sepiolite diffractions were chosen to evaluate the preferential orientation of the fibers. Supplementary Table S2 shows the values calculated for the self-supported HNT-SEP and KAOL-SEP films (at different combination ratios) compared to those obtained for the raw clay materials. The intensity ratios for the neat powder samples agree with previous literature (Ruiz-Hitzky et al., 2011a; Pasbakhsh and Churchman, 2015) and reveal a random orientation of the clay particles. However, all the intensity ratios obtained for the prepared films show significant higher values so reflecting, as suggested by SEM images, a disposition of fibers, sheets and nanotubes parallel to the plane of the film resulting in the intensification of the (100) diffractions. Moreover, it is interesting to observe the change of these parameters with the films’ composition. Thus, for the kaolinite/sepiolite system the optimal sheets orientation occurs when the kaolinite content is the 50% w/w, while for sepiolite, the fibers alignment is better as higher is the concentration of sepiolite. In contrast, for the HNT-SEP system, the film with less amount of sepiolite (50% w/w) shows the major order in sepiolite fibers, which also matches the optimal orientation of halloysite nanotubes.

**FIGURE 8:**
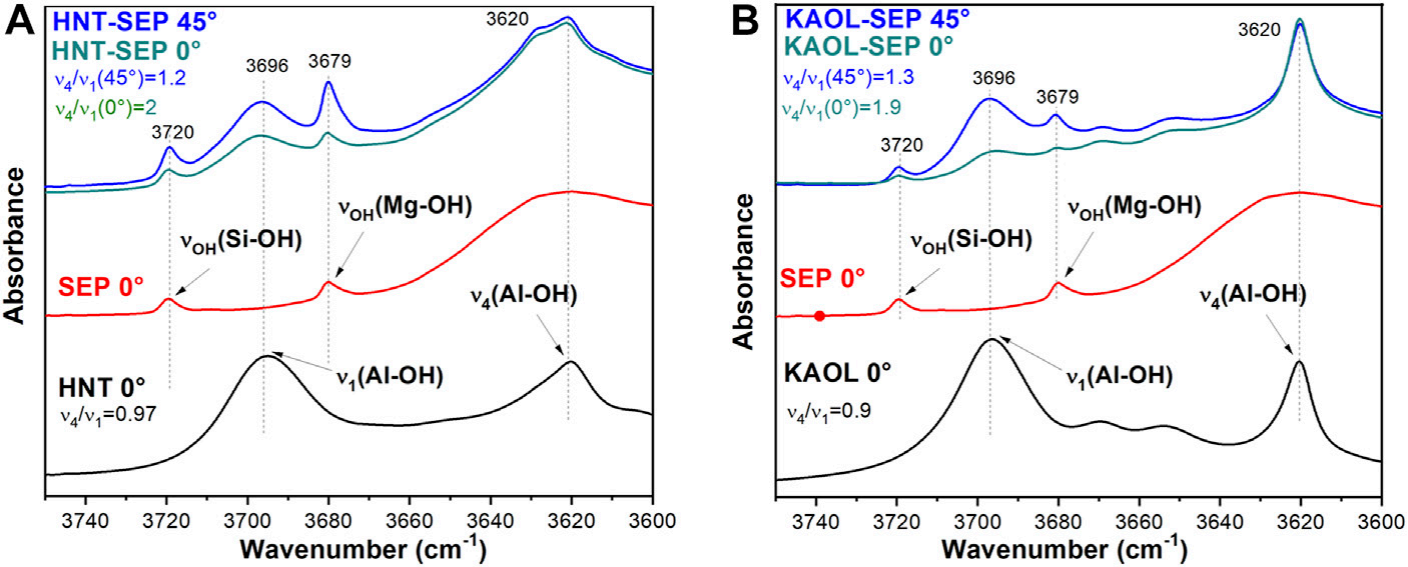
FTIR spectra of **(A)** HNT-SEP 1:2 film at 0° (green) and 45° (blue), pure halloysite (black) and sepiolite (red), and **(B)** KAOL-SEP 1:2 film at 0° (green) and 45° (blue), pure kaolinite (black) and sepiolite (red).

Additional information on the orientation of kaolinite sheets and halloysite nanotubes in the sepiolite network can be obtained through the dichroic effects observed in certain IR bands ascribed to the O-H vibration modes of the silicates. Figure 8 shows the FTIR spectra (3,750–3,600 cm^−1^ region) of pressed powders of the neat clays recorded with the beam perpendicular to the sample holder (0°), and of HNT-SEP 1:2 and KAOL-SEP 1:2 films recorded at the same orientation (0°) and after tilting the holder 45° with respect to the incidence beam (45°). The spectra of the films show clearly the typical bands associated with the sepiolite O-H stretching vibration modes of Si-OH and Mg-OH groups at 3,720 and 3,679 cm^−1^, respectively), as well as the O-H stretching vibrations of Al-OH groups centered at 3,696 and 3,620 cm^−1^. The first of these two last bands is associated with the in-plane vibration of aluminol groups with a transition moment nearly perpendicular to the (001) plane of the aluminosilicate layer (*ν*_1_) while the band at 3,620 cm^−1^ is the anti-phase vibration with a transition moment lying in the (001) plane (*ν*_4_). When comparing the spectra of the pure clays with the HNT-SEP (Figure 8A) and KAOL-SEP (Figure 8B) films at 0° position, the bands appear without significant changes in their frequency positions with respect to the starting materials, but their relative intensity is modified. In pure kaolinite and halloysite the relative intensity of *ν*_4_ and *ν*_1_ bands (*ν*_4_/*ν*_1_ ratio) is 0.9 and 1, respectively, while for the HNT-SEP 1:2 and KAOL-SEP 1:2 films rises to 2.0 and 1.9, respectively. This observation can be explained as an effect of the strong orientation of nanotubes and nanoplatelets on the plane of the film that provokes an interaction between dipole moment variation and the electric field of the beam mostly destructive. When the sample holder is tilted at 45° with respect to the incident beam, the polarization vector of the *ν*_1_ vibrations has a more constructive interaction, resulting in an increase of the intensity of the associated band and a decrease in the *ν*_4_/*ν*_1_ ratio to 1.2 and 1.3 for HNT-SEP 1:2 and KAOL-SEP 1:2 films, respectively. The bands at around 3,720 and 3,680 cm^−1^ in the films, are ascribed to the O-H stretching vibration modes of Si-OH and Mg-OH groups of the sepiolite structured. These bands are also dichroic in the films, confirming the existence of a preferential orientation of the silicate fibers (Yariv and Lapides, 2008).

To prove a potential application of this type of films, the retention of the characteristic loading capacity of HNT was evaluated with a test based on the adsorption of DMSO vapors. In the FTIR spectra of the HNT-SEP 1:5 film after exposition to DMSO vapors (Supplementary Figure S4A), it is clearly observed the presence of two new bands at 3,567 and 3,503 cm^−1^. These bands can be ascribed to O-H stretching vibration modes of water molecules associated with the DMSO (Frost et al., 1998; Yariv and Lapides, 2008). Moreover, the band at 3,696 cm^−1^ in the untreated HNT-SEP 1:5 film ascribed to the *ν*_1_ O-H vibration of halloysite aluminol moves to lower frequency, appearing at 3,663 cm^−1^ in the treated film. This shift could be indicative of formation of H bonds between the –OH groups in the halloysite lumen and the S=O group of the DMSO molecules (Kloprogge, 2017). The band at 3,620 cm^−1^ associated with the *ν*_4_ vibration (Supplementary Figure S4A) remains in its characteristic frequency as these inner OH groups located between tetrahedral and octahedral sheets of the aluminosilicate structure cannot interact with other molecules. Another relevant observation is the disappearance of the band at 3,720 cm^−1^ associated with the O-H stretching of sepiolite silanol groups indicating they are also in interaction with DMSO molecules. In this case, the band at 3,679 cm^−1^, associated with the O-H stretching of structural Mg-OH is slightly shifted towards higher wavenumbers, indicating a stronger interaction of DMSO with sepiolite. The XRD pattern of the HNT-SEP 1:5 film after exposition of DMSO (Supplementary Figure S4B) does not show differences with respect to the untreated HNT-SEP 1:5 film, confirming DMSO molecules interact almost exclusively with the external surface of sepiolite fibers and with the inner surface of HNT but without intercalation in this later silicate.

### Sepiolite-LDH Nanoarchitectures

The possibility to assemble sepiolite nanofibers and LDH platelets at the nanoscale could be also of interest to produce nanoarchitectured materials that display combined properties of each type of nanoparticles with some synergistic effects. In this way, we have developed and patented several years ago a methodology based on the co-precipitation of the LDH in presence of sepiolite, whose external silanol groups act as nucleating points for the growth of LDH nanoparticles (Ruiz-Hitzky et al., 2011b). Following this synthetic route, diverse LDH-sepiolite materials have been developed, which can be used as simultaneous cation and anion adsorbents (Gómez-Avilés et al., 2016), nanofillers of Nafion membranes with improved proton conductivity (Charradi et al., 2019) or as controlled delivery systems of herbicides (Rebitski et al., 2019). A thermal treatment of such heterostructure could also transform the LDH hydroxylated structure into well dispersed mixed oxide on the fibrous clay of interest in catalysis (Gómez-Avilés et al., 2016). It is also possible to produce the re-construction of the LDH from their corresponding layered double oxide phases (LDO) in the presence of sepiolite, but in the resulting sepiolite-LDH systems the LDH particles are not strongly linked to the sepiolite fibers (Gómez-Avilés et al., 2016). The present work introduces an alternative approach to the development of sepiolite-LDH nanostructured materials from both starting components, sepiolite and LDH prepared as water dispersions under ultrasound irradiation that can be further processed, for instance to produce homogeneous self-supported films.

**FIGURE 9:**
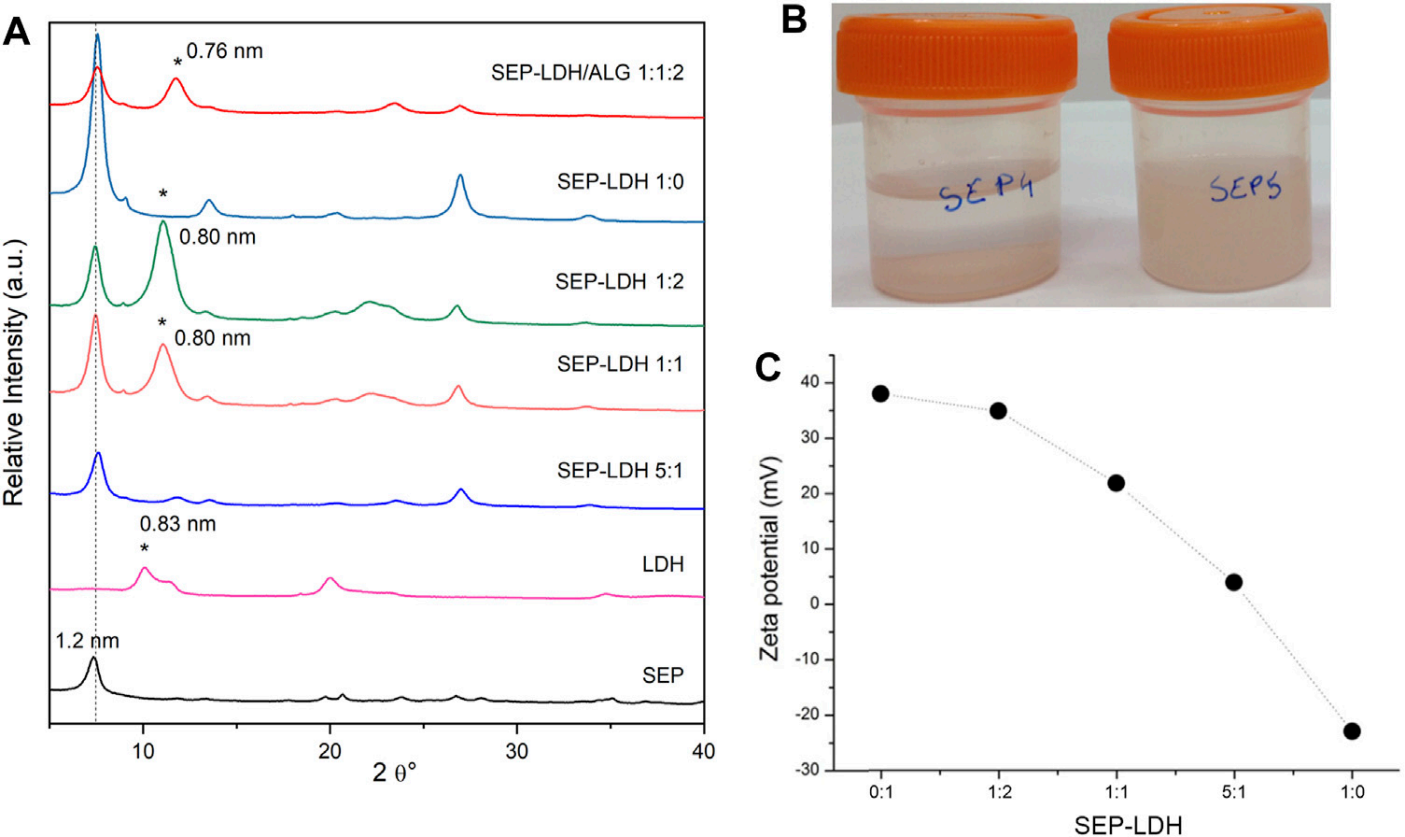
**(A)** Powder XRD patterns of starting components and sepiolite-LDH and sepiolite/LDH/alginate films of various compositions (*peak of LDH). **(B)** Aspect after 3 months of its preparation of sepiolite/LDH starting dispersions without (left) and with (right) ultrasonication (20 mg sepiolite/20 mg MgAl-NO_3_ in 10 ml of water, US 1 kJ), and **(C)** evolution of the Z potential for the sepiolite/LDH sonicated dispersions.

For this study, a MgAl-NO_3_ LDH was first prepared, whose structure is confirmed by XRD (Figure 9), the broadening of the 00l reflection indicating the presence of a side MgAl-CO_3_ phase due to contamination by atmospheric CO_2_ during the synthesis. TEM images (Supplementary Figure S5) confirm the synthesis of rather well dispersed nanoparticles displaying a size lower than 100 nm while a slightly higher particle size (around 230 nm) is determined by DLS attributed to hydrodynamic radius and partial aggregation of the particles. Then, various dispersions of sepiolite and the LDH were prepared using various proportions of each component (Table 1). Without ultrasonication, a syneresis effect is observed after few minutes (Supplementary Figure S6). Interestingly, by applying ultrasonication treatment at 1 kJ, the resulting SEP-LDH dispersions show an excellent stability (Figure 9B). Syneresis effects are not observed even for 3 months, the dispersions maintaining an elevated viscosity. Such observation evidences that ultrasonic treatment, which induces sepiolite fiber disaggregation (Supplementary Figure S5), hampers the heterocoagulation phenomenon usually observed due to charge neutralization of two types of oppositely charged particles.

Zeta potential measurements confirmed the negatively charge surface of sepiolite while a positive value of zeta potential is obtained for MgAl-NO_3_, in good agreement with positively charged layers. The zeta potential decreased with increasing sepiolite amount due to the adsorption of the oppositely charged LDH nanoparticles (Figure 9C). Charge neutralization occurred approximatively for a sepiolite:LDH ratio of 75:25, where the overall charge of the particles was zero. Moreover, the adsorption continued beyond this point and overcharging was observed at higher sepiolite doses. Such change in the sign of the surface charge is typical of systems containing colloidal particles and oppositely charged polyelectrolytes or multivalent ions (Szilagyi et al., 2014) and has already been reported in the literature for similar heterocoagulated clay-latex particle (Kobayashi et al., 2013; Veschambres et al., 2016). Clearly, these results confirmed the favorable electrostatic interaction between the two inorganic matrices.

**FIGURE 10:**
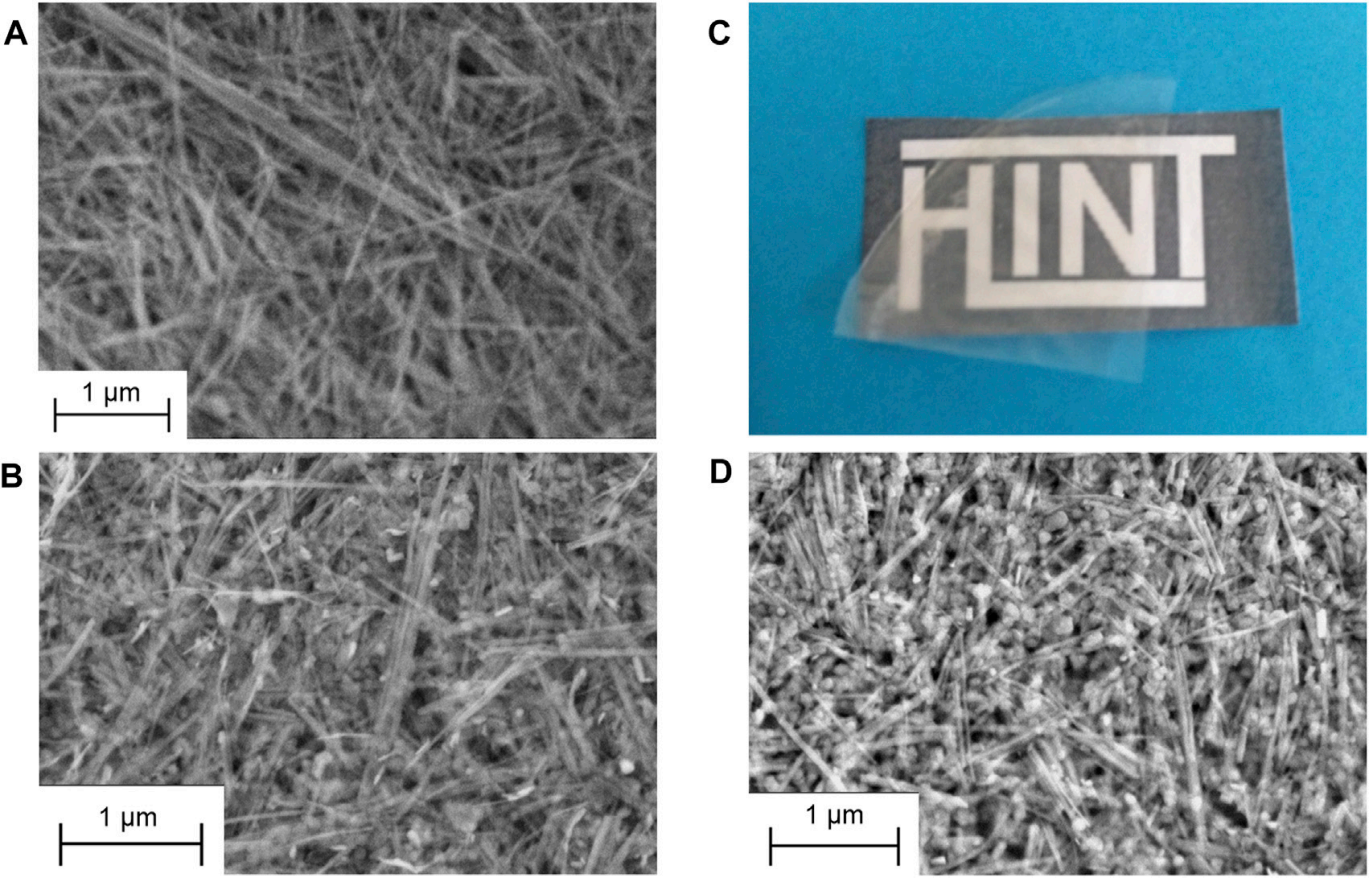
FE-SEM images of SEP-LDH films prepared with dispersions of the components at ratio of **(A)** 1:0 and **(B)** 1:1; and aspect of a film obtained after casting and drying of a SEP-LDH/ALG 1:1:2 dispersion observed **(C)** at the eye and **(D)** by FE-SEM.

The high stability of the sepiolite and LDH dispersions allows the easy preparation of highly homogeneous films by vacuum filtration and drying. The XRD patterns of resulting SEP-LDH self-supported films of various compositions (Figure 9A) evidence the presence of both crystalline phases with relative intensity of the main reflection in good agreement with the amount involved for each of them. The FE-SEM images of dried dispersions of pure sepiolite (SEP-LDH 1:0) compared to SEP-LDH 1:1 evidence a good dispersion of the LDH nanoparticles within the sepiolite fibers (Figures 10A,B).

An interesting issue for these SEP-LDH heterostructures is the possibility to further combine them with biopolymers to produce bionanocomposites displaying interesting properties. Thus, the good colloidal stability of the sepiolite-MgAl-NO_3_ nanoparticles dispersions has been used to produce, in an easy way, homogeneous systems with alginate. Since it was previously reported that sepiolite dispersions were not stable when alginate is added before the US treatment (Ruiz-Hitzky et al., 2016), the alginate addition was carried out once the inorganic phase dispersion is prepared to avoid syneresis effect. After casting and drying the three-components dispersion, self-standing transparent SEP-LDH/ALG films with homogeneous aspect are produced (Figure 10C). FE-SEM images of the SEP-LDH/ALG 1:1:2 bionanocomposite (Figure 10D) show a similar morphology than the SEP-LDH 1:1 heterostructured material (Figure 10B), evidencing that the alginate addition did not induce modification of the distribution of LDH nanoparticles assembled on the surface of the sepiolite fibers. Moreover, the possibilities to further intercalate or immobilize functional organic or biological species on these type of systems open a panoply of potential applications from uses in wastewater treatment through adsorption process, environment monitoring and biosensors development, and others.

## Concluding Remarks

Sonomechanical treatment (i.e., ultrasounds irradiation or ultrasonication) of aqueous dispersions of sepiolite produces high viscosity hydrogels attributed to the efficient disaggregation of the silicate fibers that are initially present as bundles. These hydrogels can be transformed into low-density xerogels by application of diverse drying procedures, including air-drying of filtered or centrifuged samples, freeze-drying and spray-drying techniques. The resulting xerogels exhibit textural behaviors and rheological properties that depend on the adopted experimental conditions for their preparation, and especially on the selected drying procedure. Some characteristics of these xerogels are comparable to commercially available sepiolite of rheological grade, being able to be re-dispersed in water under high-speed shear, generating very stable and viscous hydrogels and minimizing the syneresis phenomena usually observed in water dispersions of untreated sepiolite.

Sepiolite hydrogels are colloidal systems used to prepare nano- and micro-architectured solids. In this context, we have applied colloidal routes to prepare new heterostructured functional materials, by controlled incorporation of clay-related solids of diverse topology and composition, such as respectively 2D and 1D kaolinite and halloysite aluminosilicates, as well as to the 2D synthetic Mg,Al-layered double hydroxides (LDH). The essential role of fibrous dispersions of sepiolite is its ability to promote the formation of self-supported films inducing a preferential orientation of the clay particles.

The procedures studied here, based on sepiolite gels, could be used to develop new full clay-based materials of different topology since they could include diverse components such as smectites and vermiculites, talc and pyrophyllite or various types of micas.

In addition, other organic or inorganic nanoparticles could be also introduced to add functional properties. It should be remarked the possibility to assemble these heterostructures with polymers. As mentioned in this work, we have successfully combined these inorganic materials with water-soluble polymers, such as alginate, showing a promising way to prepare new functional hybrid materials that could be shaped as films, monoliths or foams. Likewise, their properties as micro- and mesoporous materials should be explored, which could be of particular interest in their application, for example, as gas separation membranes, as heterogeneous catalysts or as support for bioactive species.

## Data Availability

The original contributions presented in the study are included in the article/Supplementary Material, further inquiries can be directed to the corresponding author.
