# Supplementary material for: Sepiolite-Hydrogels: Synthesis by Ultrasound Irradiation and Their Use for the Preparation of Functional Clay-Based Nanoarchitectured Materials
Source: Front Chem. 2021 Aug 13;9:733105. doi: 10.3389/fchem.2021.733105 (PMC8414812; doi:10.3389/fchem.2021.733105)
Supplement: Supplementary file 1 [file DataSheet1.DOCX]

Supplementary Material

**Supplementary Table 1.** Characteristics of the sepiolite clays used.

| **Clay** | **Origin** | **Chemical composition (%)** | | | | | **Particle size (µm)** | **Water content (%wt)** |
| --- | --- | --- | --- | --- | --- | --- | --- | --- |
|  |  | SiO_2_ | MgO | CaO | Al_2_O_3_ | Fe_2_O_3_ |  |  |
| **Sepiolite 30-60** | TOLSA SA, (Tagus Basin, Spain) | 60.5 | 23.8 | 0.5 | 2.4 | 0.2 | 25.0-60.0 | 12.5 |
| **Sepiolite-1** | SEPIOLSA, (Tagus Basin, Spain) | 60.1 | 25.4 | 1.7 | 1.2 | 0.4 | 35.5−11.1 | 12.5 |
| **Sepiolite-2** | ZAFER MINING CO., (Balıkesir, Turkey) | 60.5 | 20.9 | 0.3 | 2.5 | 1.1 | 30.2−11.6 | 17.9 |

**Supplementary Table 2.** Intensity ratio of the main XRD reflection peaks determined from the diffractograms of sepiolite (SEP), halloysite (HNT) and kaolinite (KAOL) in HNT-SEP, KAOL-SEP films and neat clays.

| **Sample** | **Intensity ratio** | | | |
| --- | --- | --- | --- | --- |
|  | **SEP (200)/(110)** | **SEP (400)/(110)** | **HNT or KAOL (100)/(020)** | **HNT or KAOL (200)/(020)** |
| **HNT-SEP 1:5** | 0.3 | 4.5 | 0.6 | 2.3 |
| **HNT-SEP 1:2** | 0.5 | 1.5 | 3.3 | 2.1 |
| **HNT-SEP 1:1** | 0.8 | 8.2 | 1.0 | 3.0 |
| **HNT** | - | - | 0.5 | 0.5 |
| **SEP** | 0.07 | 0.15 | - | - |
| **KAOL** | - | - | 4.3 | 4.2 |
| **KAOL-SEP 1:5** | 1.6 | 6.7 | 17.0 | 9.7 |
| **KAOL-SEP 1:2** | 1.0 | 4.1 | 19.0 | 13.0 |
| **KAOL-SEP 1:1** | 0.2 | 0.6 | 26.0 | 14.0 |

From the values in Supplementary Table 2, it can be deduced that the intensity ratio of (100)/(020) and (200)/(020) reflections in the KAOL-SEP films increases, respectively, from 17 to 26 and 9.7 to 14 as the content of kaolinite increases, reaching the optimal sheets orientation when the kaolinite is the 50%w/w. The same trend was found for the (200)/(110) and (400)/(110) parameters. In fact, these values rise from 0.2 to 1.6 and 0.6 to 6.7 as the content in sepiolite increases from 50%w/w to 85%w/w, suggesting that the fibers alignment is better as higher is the concentration of sepiolite. On the other side, the interpretation of the parameters calculated for the HNT-SEP films reveals that the film with less amount of sepiolite (50% w/w) shows the major order in sepiolite fibers. In fact, the (200)/(110) and (400)/(110) ratios reach the higher values of 0.8 and 8.2. Furthermore, taking into account the higher values of the (100)/(020) and (200)/(020) parameters, the HNT-SEP 1:2 and HNT-SEP 1:1 films seem to show the optimal orientation of halloysite nanotubes.





**Supplementary Figure 1.** N_2_ adsorption-desorption isotherms at 77 K of the sepiolite xerogels samples.

**
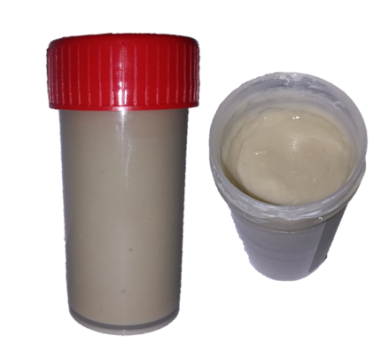
**

**Supplementary Figure 2.** Macroscopic aspect of 6 wt% colloidal dispersion in water of XG-FV after 10 years.


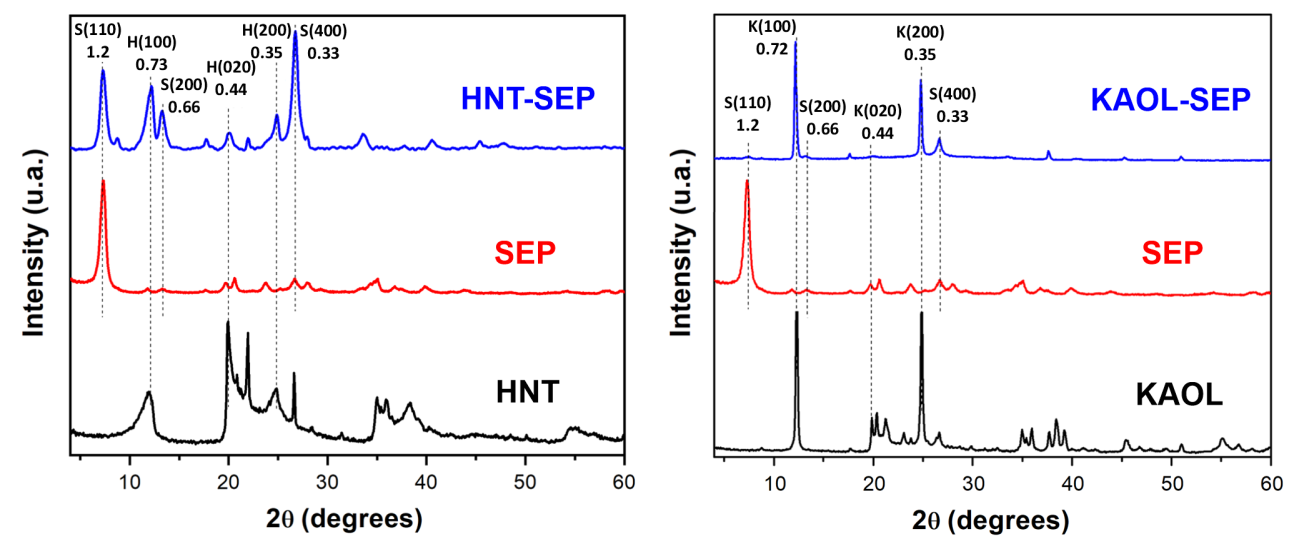


**Supplementary Figure 3.** XRD patterns of starting sepiolite (SEP) halloysite nanotubes (HNT) and kaolinite (KAOL) and the self-supported films HNT-SEP 1:2 and KAOL-SEP 1:2.


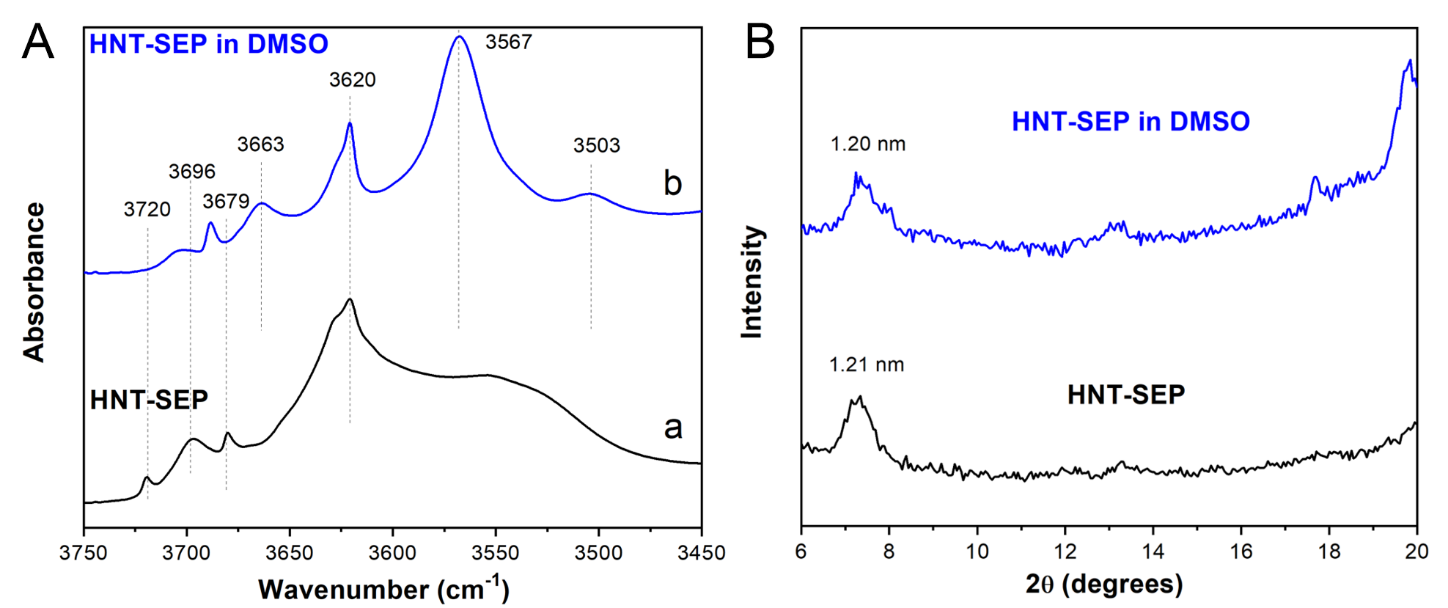


**Supplementary Figure 4.** **(A)** FTIR spectra and **(B)** XRD patterns of HNT-SEP 1:5 film before and after exposition to DMSO vapors.


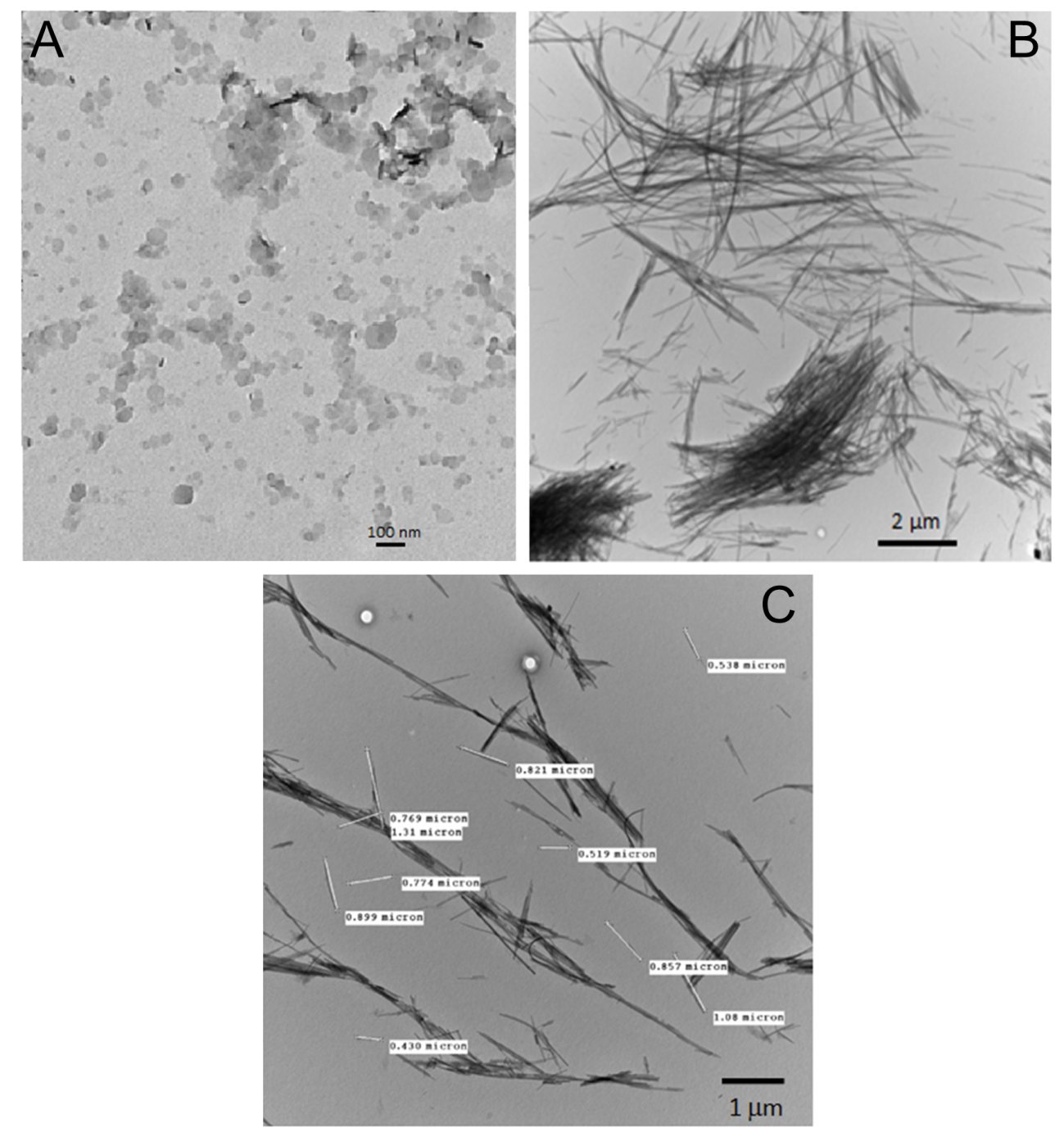


**Supplementary Figure 5.** TEM images of **(A)** the prepared MgAl-NO_3_ LDH; and of the natural sepiolite particles **(B)** before and **(C)** after ultrasonication.


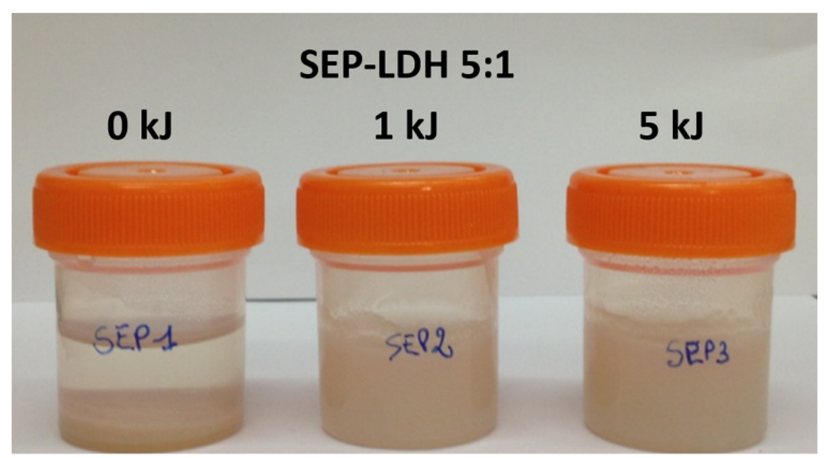


**Supplementary Figure 6.** Aspect of sepiolite-LDH dispersions (100 mg sepiolite/20 mg MgAl-NO_3_ in 10 mL of water) prepared at different ultrasonication energy, a few minutes after their preparation.
